# Leprosy in the United States and British Provinces

**Published:** 1896-06

**Authors:** 


### LEPROSY IN THE UNITED STATES AND BRITISH PROVINCES.

Another leper, this time American born and 54 years of age, has joined the colony at North Brother Island, New York City. He applied for treatment of a skin disease at the Presbyterian Hospital clinic, where leprosy was promptly diagnosed. He had resided in New York for sixteen years, and for a long time was a sea-going cook. The colony above referred to consists now of a negro, a Chinaman, an Italian and this American. Along with this announcement by the lay press of March 5th came recent government reports from Canada, which state that leprosy is increasing in New Brunswick and Nova Scotia. There are now a number of cases of leprosy in Cape Breton, N. B., and others scattered all over the province. There is one district in New Brunswick, Port Hood, where it has existed for years among the poor fishing settlements. Tradition traces it back to a French vessel, *L. Indienne*, which had been trading in the Levant and was wrecked at the mouth of the Miramiction River in 1758. It is said that she had on board some Lascar or other Oriental sailors who were lepers. Clothing belonging to some of these men was distributed among the fishermen. The wreck took place in the fall, and the crew associated with the fishermen all winter. Since then leprosy in a dormant way has been present among the French settlers in this region. When the Acadians were expelled by the English in 1765 and were removed to Louisiana it is said that cases of leprosy were taken with them, for there have always from that date been cases of leprosy in the neighborhood of New Orleans, and a leper hospital was established to care for those afflicted only a few years later, in 1785; but this hospital has been discontinued. In Nova Scotia there have been a few cases of leprosy, but confined to the families of certain Scotch and Irish settlers.

A Norwegian colony in Minnesota and the leper hospital in San Francisco since 1871 show, to say the least, that the Western Hemisphere is not without its opportunities for special study. The fact that the spread of leprosy is slow, and in some countries seems to have ceased, better enables us to trace the course of its dissemination and the conditions of its environments. Thus far it is an importation, or at least carries a foreign stamp. In countries where the spread is rapid it is believed to be both contagious and hereditary, but probably the observation is from the numerical standpoint alone. Therefore our great sister city has, or is likely yet to have, many opportunities for a study in miniature. But with the growth of personal liberty and the loose methods of charitable institutions there cannot well be many advances along the line of isolation or segregation. Meanwhile the Leprosy Investigating Committee of England, under the patronage of the Prince of Wales, has, also with its Indian connection, a wide field. This organization, it will be remembered, had its birth in the promotion of a memorial for Father Damien, a self-sacrificing sceptic to the doctrine of contagion, and to the fact that a leper had been found handling fresh meat in a London market.—*Jour. Amer. Med. Ass'n.*

